# Value of contrast-enhanced ultrasound in diagnosis and differential diagnosis of polypoid lesions of gallbladder ≥ 1 cm

**DOI:** 10.1186/s12876-022-02373-z

**Published:** 2022-07-25

**Authors:** Xiaoyue Zhang, Shaoshan Tang, Liping Huang, Hong Jin, Yijiao Wang, Yao Wang, Zhan Liu, Chunyu Lu

**Affiliations:** Department of Ultrasound, Shengjing Hospital of China Medical University, Sanhao Street No. 36, Heping District, Shenyang, 110004 Liaoning Province China

**Keywords:** Gallbladder, Ultrasonography, Neoplasms, Contrast media, Diagnostic accuracy

## Abstract

**Objectives:**

To evaluate the usefulness of Contrast-enhanced ultrasound (CEUS) in the diagnosis and differential diagnosis of Polypoid lesions of gallbladder (PLGs) ≥ 1 cm.

**Methods:**

A prospective analysis was performed on 180 patients with PLGs ≥ 1 cm. 175 cases were confirmed by pathological diagnosis and the remaining were confirmed by other imaging findings. The characteristics of lesions on conventional Ultrasonography (US) and CEUS were recorded.

**Results:**

Significant differences were observed in enhancement patterns between benign and malignant PLGs during both arterial (P < 0.001) and venous phases (P < 0.001). The malignant lesions typically yielded a “fast-in and fast-out” enhancement pattern. There was no significant difference in Arrival time (AT) between malignant and benign PLGs. If we consider wash-out time ≤ 40 s as a diagnostic standard for malignant lesions, the sensitivity, specificity, and accuracy were 88.24%, 85.62%, and 86.11%, respectively. Destruction of the Gallbladder (GB) wall was a particularly important indication of malignant PLGs, and the sensitivity, specificity, and accuracy were 93.33%, 92.12%, and 92.22%, respectively. The accuracy of CEUS in the diagnosis of PLGs, as well as malignant and benign lesions, was 92.22%, 92.47%, and 91.17%, respectively.

**Conclusions:**

The “fast-in and fast-out” enhancement pattern, hyper-enhancement in comparison to the GB wall in the arterial phase, wash-out time ≤ 40 s, GB wall destruction, and hepatic parenchymal infiltration are the characteristic findings of malignant PLGs. Besides, CEUS provides a valuable reference to classify some of the benign lesions.

## Key points

1. CEUS provides greater differential diagnostic confidence to malignant and benign PLGs.
2. The classification of some benign lesions can be given greater confidence with the help of CEUS.
3. CEUS may reduce the need for surgery.

## Introduction

Polypoid lesions of the gallbladder (PLGs) refer to the lesions derived from gallbladder (GB) wall that protrude into GB lumen. They are a relatively common disease, but with a poor prognosis. Generally, laparoscopic or open cholecystectomy is used for PLGs > 1.0 cm [1, 2]. However, most of these are benign (68.6%) [3], and some of malignant lesions infiltrate the GB wall or the adjacent hepatic tissue can’t be detected before the operation because of the limitation of some imaging examinations in the diagnosis of early lesions [4, 5]. Therefore, accurate preoperative diagnosis is particularly important in selecting treatment options, determining the scope of the operation, and avoiding unnecessary surgery.

Conventional ultrasonography (US) is considered the first-line imaging examination for GB diseases. However, there are inevitable limitations associated with the technique, such as its utility in differentiating motionless sludge from true neoplasms, detection of low-velocity blood flow, etc. Some studies have shown that contrast-enhanced ultrasound (CEUS) can overcome the limitations associated with conventional US and increase the diagnostic accuracy for GB diseases (the accuracy of CEUS and US were 95.2% and 88.6%, respectively) [6, 7]. Conventional US combined with CEUS can greatly improve the diagnostic accuracy of GB diseases and provide diagnostic complementary information for computed tomography (CT) and magnetic resonance imaging (MRI) [8, 9]. However, the significance of the characteristics in CEUS in diagnosing GB diseases is still controversial and needs more research to summarize.

This study aims to evaluate the diagnostic yield of CEUS for the differential diagnosis of PLGs ≥ 1 cm.

## Materials and methods

### Study population

180 patients diagnosed with PLGs ≥ 1 cm in Shengjing Hospital of China Medical University by conventional US were selected, including 63 (35%) male and 117 (65%) female patients with a mean age of 46.8 years old (SD ± 13.1 years old). Then, all patients underwent CEUS after signing the written informed consent. The patient cannot meet one of the exclusive criteria (severe cardiovascular or cardiopulmonary disease; pregnant or lactating females; with a history of allergies, especially those allergic to contrast agents) to ensure the safety of CEUS examination. Except for five cases confirmed by magnetic resonance cholangiopancreatography (MRCP) or contrast-enhanced computer tomography (CECT), the remaining 175 cases were all confirmed by pathological examination after surgery which was performed within one week after the CEUS.

### Equipment and contrast agent

Two kinds of US systems were used: Toshiba Aplio 500 and GE LogiQ 9. The central frequency of the broadband convex array probe was 3.5 MHz and the mechanical index (MI) ranged from 0.08 to 0.12. The second-generation blood pool contrast agent, SonoVue (Bracco, Milan, Italy), consisting of phospholipid-stabilized shell microbubbles filled with sulfur hexafluoride gas, was used. In each case, a 2.4 ml bolus injection of SonoVue was administered via the antecubital vein, immediately followed by a 5 ml bolus of saline solution. If the CEUS result was unsatisfactory, the procedure was repeated with a time interval of 10 min. The images were stored in DICOM files.

### Examinations and image reading

Abdominal US and CEUS were performed by experienced sonographers, who worked for > 5 years with CEUS and were not involved in later data analysis. Each patient fasted for at least 8 h before the examination.

Before CEUS was performed, each patient underwent conventional US to thoroughly check the GB and liver tissue. The lesion’s location, size, shape, echo characteristics, boundary, width at the base, and blood flow within the lesion were recorded. The continuity of the GB wall and echo changes of the surrounding liver were also observed.

After the conventional US, CEUS was then performed. Each patient was asked to hold a deep breath for the entire procedure, or hold the breath for the first 30 s, to obtain the desired views. The enhancement process was divided into arterial phase (0–30 s after contrast injection) and venous phase (31–180 s after contrast injection). The extent of enhancement was classified into hypo-, iso-, hyper-, and non-enhancement, with reference to the surrounding normal GB wall. The contrast arrival time (AT) of the lesions and wash-out time were recorded by observing the entrance and exit of contrast medium in the CEUS image. Because in the process of CEUS, affected by the patient's breathing, the lesions move greatly, which is not suitable for the time-contrast curve. After 120 s, the transducer was moved to examine the entire hepatic parenchyma, especially the adjacent tissue, to exclude liver infiltration or metastases. For those patients with multiple lesions, the largest lesion was generally selected for the study. The whole process of CEUS was recorded and stored in the hard disk of the US machine to facilitate later analysis. Intraoperative and pathological findings were collected and compared with those of conventional US and CEUS images.

### Statistical analysis

All statistical analyses were carried out using the SPSS 17.0 software (SPSS, Chicago, IL, USA). Quantitative data were expressed as mean ± SD, and qualitative data were expressed as a percentage. Comparisons among qualitative data were tested using the Chi-squared test. The independent *t* test was used for comparisons between groups. Any *P* values < 0.05 were considered statistically significant.

## Results

### Pathology

Histological diagnosis revealed 29 malignant cases and 146 benign cases. The malignant cases included 19 cases of adenocarcinoma, three cases of adenosquamous carcinoma, and seven cases developed from benign lesions (one case of adenomyomatasis and six cases of adenoma). The benign cases included 100 cases of cholesterol polyps, 29 cases of adenoma, ten cases of adenomyomatosis, three cases of chronic cholecystitis, two cases of cholecystolithiasis, one case of metaplastic polyp, and one case of hyperplastic polyp. The other five cases were confirmed malignant lesions by MRCP or CECT.

### Conventional US

Among the 146 benign lesions, 77 cases (52.7%) were single and 69 cases (47.3%) were multiple; 129 cases (88.4%) had narrow bases or a thin pedicle. The mean diameter of the benign lesions was 1.33 ± 0.53 cm (range 1.0–5.8 cm), and the blood flow signal of only 14.4% of those lesions could have been detected.

Among the 34 malignant lesions, 28 cases (82.4%) were single with wide bases. The mean diameter of the malignant lesions was 3.29 ± 1.70 cm (range 1.1–7.2 cm). Blood flow was evident in 27 cases (79.4%) on CDFI. Significant differences between malignant and benign lesions were observed in diameter, number, width of the base, and blood flow signals within the lesion (all *P* < 0.05). The results are presented in Table 1.

**Table 1:** The characteristics of ALGs of different pathology types on conventional US

Final diagnosis	n	Number^a^		Size (diameter)^b^			Basement^c^		Vascularity^d^	
		Single	Multiple	< 2.0 cm	2.0—3.0 cm	> 3.0 cm	Narrow	Wide	Yes	No
Malignant lesions	34	28 (82.4%)	6 (17.6%)	7 (20.6%)	12 (35.3%)	15 (44.1%)	6 (17.6%)	28 (82.4%)	27 (79.4%)	7 (20.6%)
Benign lesions	146	77 (52.7%)	69 (47.3%)	136 (93.2%)	7 (4.8%)	3 (2.0%)	129 (88.4%)	17 (11.6%)	21 (14.4%)	125 (85.6%)
Cholesterol polyp	100	50 (50%)	50 (50%)	98 (98%)	2 (2%)	0 (0%)	96 (96.0%)	4 (4.0%)	12 (12.0%)	88 (88.0%)
Adenoma	29	17 (58.6%)	12 (41.4%)	23 (79.3%)	4 (13.8%)	2 (6.9%)	22 (75.9%)	7 (24.1%)	8 (27.6%)	21 (72.4%)
Adenomyomatosis	10	9 (90.0%)	1 (10%)	9 (90%)	1 (10%)	0 (0%)	4 (40.0%)	6 (60%)	1 (10.0%)	9 (90%)
Chronic cholecystitis	3	0 (0%)	3 (100%)	3 100%	0 (0%)	0 (0%)	3 (100%)	0 (0%)	0 (0%)	3 (100%)
Gallstone	2	1 (50%)	1 (50%)	1 (50%)	0 (0%)	1 (50%)	2 (100%)	0 (0%)	0 (0%)	2 (100%)
Hyperplastic polyp	1	0 (0%)	1 (100%)	1 (100%)	0 (0%)	0 (0%)	1 (100%)	0 (0%)	0 (0%)	1 (100%)
Metaplasic polyp	1	0 (0%)	1 (100%)	1 (100%)	0 (0%)	0 (0%)	1 (100%)	0 (0%)	0 (0%)	1 (100%)

^a^Number of the lesions showed statistically significant difference between benign and malignant lesions. χ.^2^ = 9.950, *P* = 0.002

^b^Diameter of the lesions showed statistically significant difference between benign and malignant lesions. t = 11.706, *P* = 0.000

^c^Stalk width of the lesions showed statistically significant difference between benign and malignant lesions. χ.^2^ = 73.537, *P* = 0.000

^d^Detection of blood floor within the lesions showed statistically significant difference between benign and malignant lesion. χ.^2^ = 59.634, *P* = 0.000

### CEUS

1. Characteristics of PLGs of different pathology types on CEUS

The enhancement patterns of PLGs on CEUS images are shown in Table 2.

**Fig. 1:**
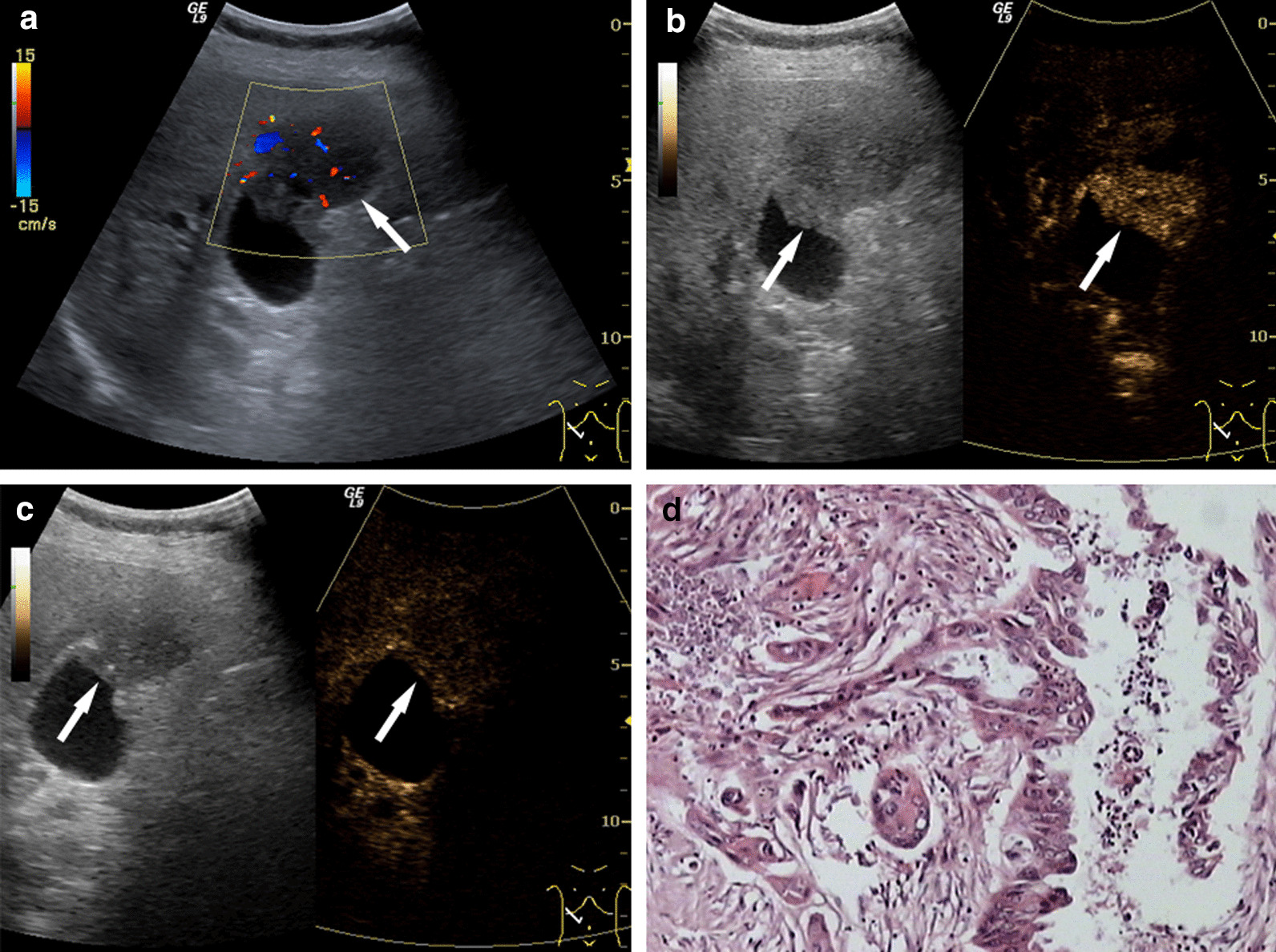
Gallbladder carcinoma. **a** Ultrasonography (US) showed a hypoechoic tumor, 5.2 × 4.3 cm in size with an obscure boundary. The blood flow can be detected within the tumor on Color Doppler flow imaging. **b**, **c** On contrast-enhanced ultrasound (CEUS), the lesion showed heterogeneous enhancement at 18 s in the arterial phase (**b**) and hypo-enhancement in comparison to the normal gallbladder (GB) wall at 34 s in the venous phase (**c**). The central non-enhancement area was presented through the whole CEUS process. **d** Postoperative pathological results: moderately to poorly differentiated GB carcinoma with infiltration of the GB wall

**Fig. 2:**
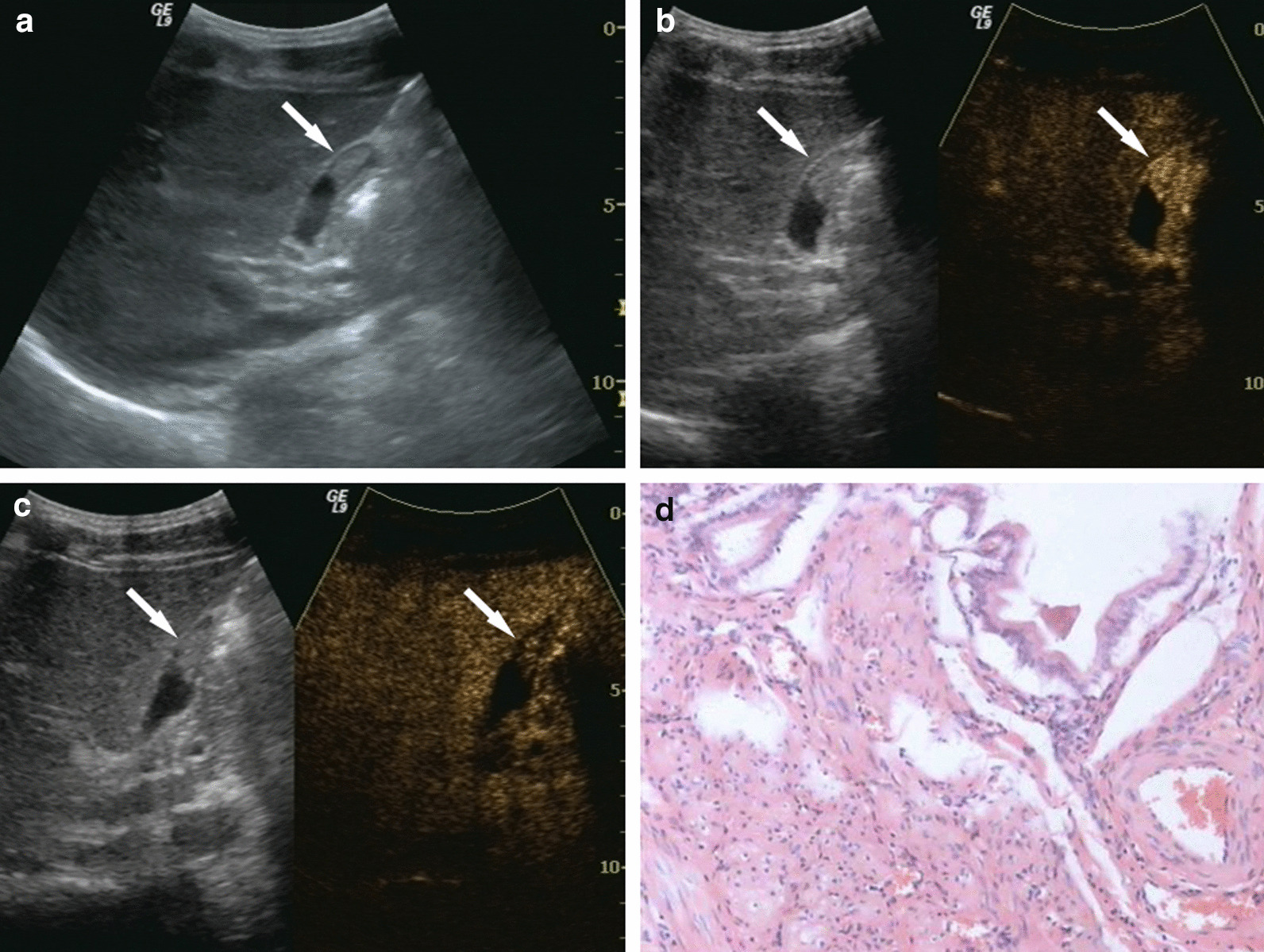
Gallbladder cholesterol polyp. **a** Ultrasonography (US) showed an isoechoic lesion, 1.4 × 0.6 cm in size. **b** On contrast-enhanced ultrasound (CEUS), the lesion showed synchronous enhancement with the gallbladder (GB) wall at 14 s in the arterial phase, and iso-enhancement in comparison to the normal GB wall. **c** As time passed, the contrast within the lesion showed simultaneous wash-out with the GB wall. **d** Postoperative pathological results: GB cholesterol polyps

**Fig. 3:**
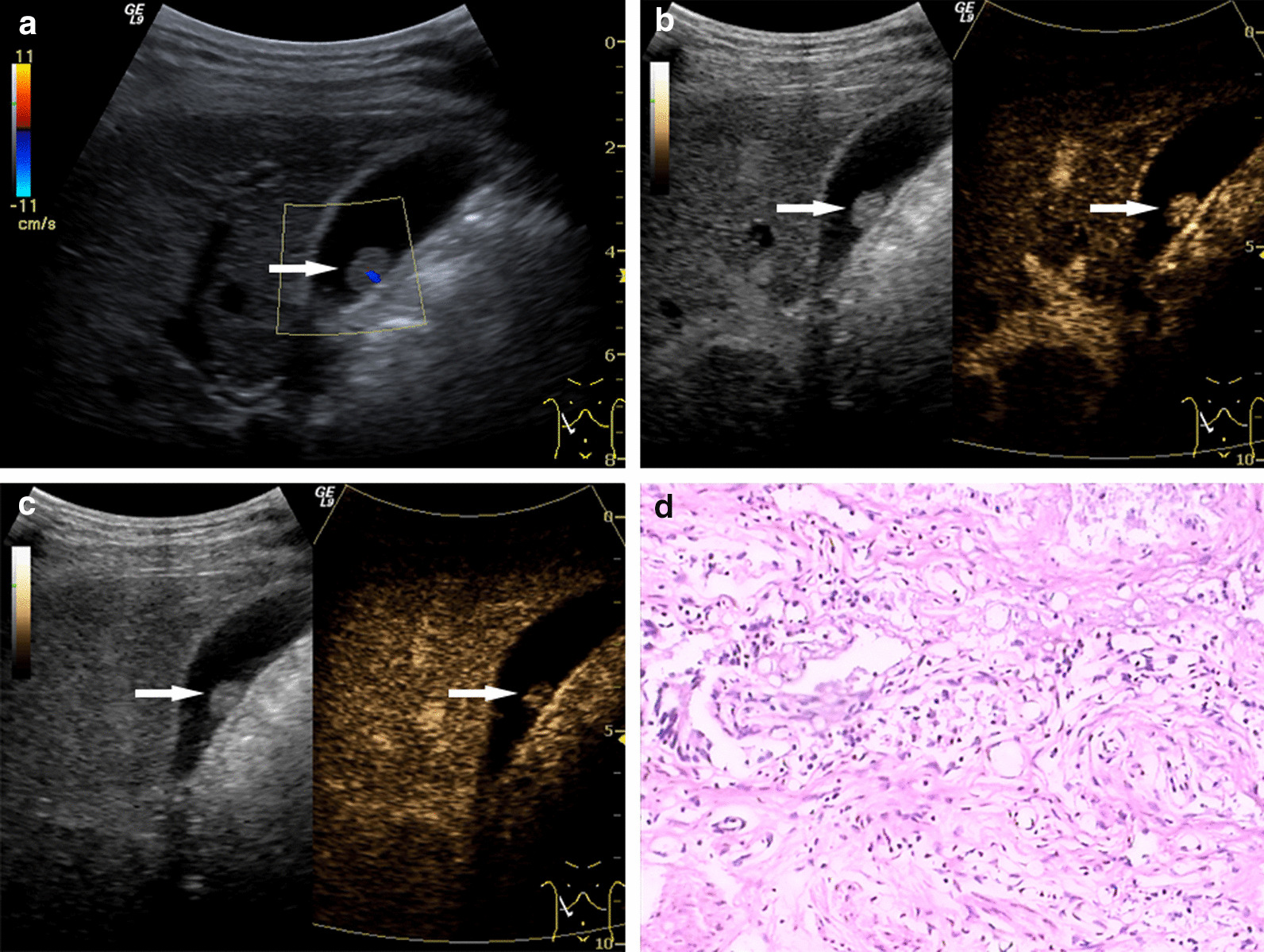
Gallbladder adenoma. **a** Ultrasonography (US) showed an isoechoic lesion, 1.0 × 0.7 cm in size, with detectable blood flow on Color Doppler flow imaging. **b**, **c** On contrast-enhanced ultrasound (CEUS), the lesion showed synchronous enhancement with the gallbladder (GB) wall at 18 s in the arterial phase (**b**) and gradually showed hypo-enhancement at 38 s, slightly in advance of the GB wall (**c**). **d** Postoperative pathological results: GB adenoma

**Fig. 4:**
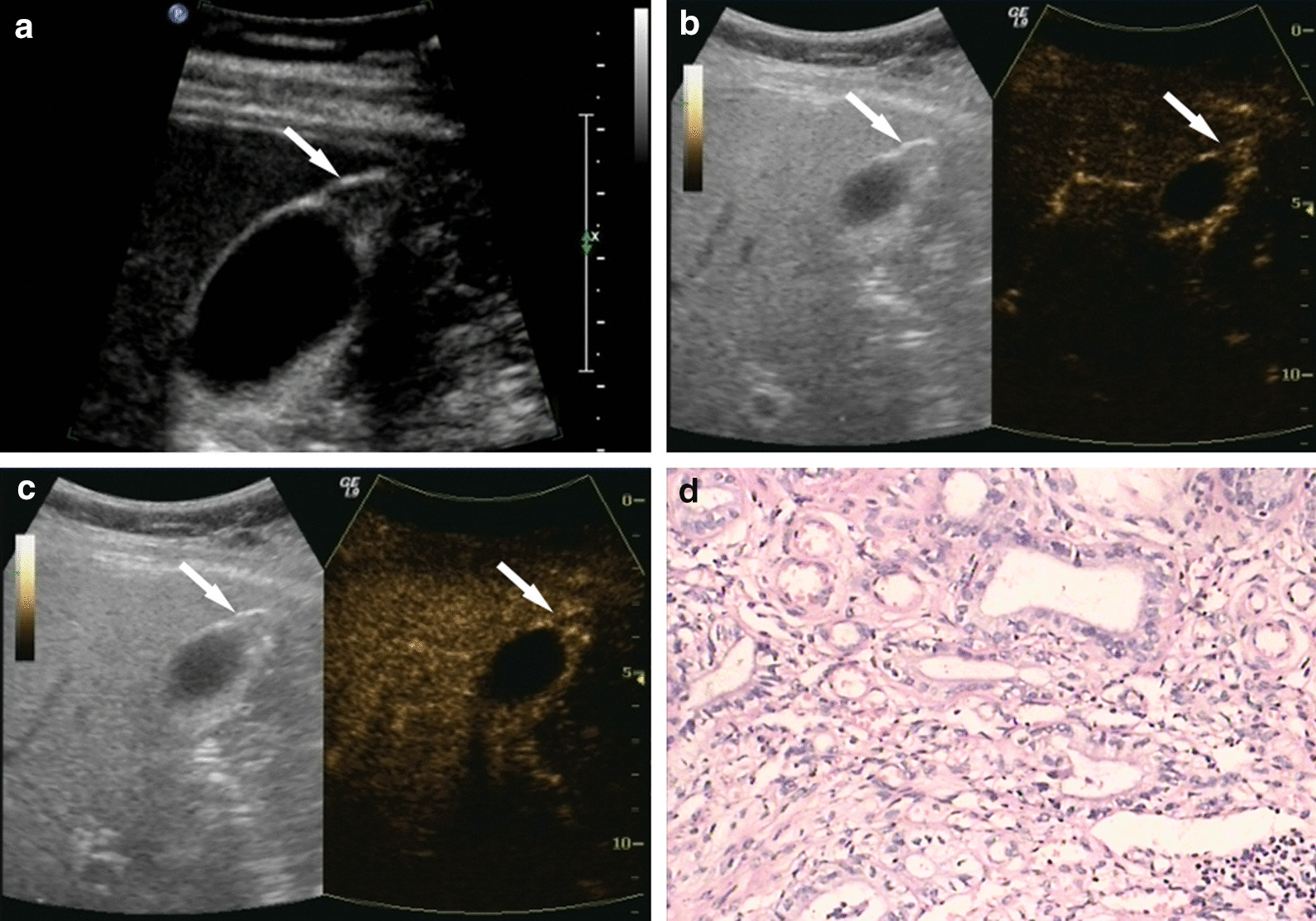
Gallbladder adenomyomatosis.** a** Ultrasonography (US) showed limited thickening at the bottom of the gallbladder (GB) wall, 1.3 × 1.0 cm in size. **b** On contrast-enhanced ultrasound (CEUS), the lesion showed heterogeneous iso-enhancement at 16 s in the arterial phase. **c** The contrast agent within the lesion then gradually showed wash-out with the GB wall. Honeycomb-like non-enhancement was observed during the entire CEUS procedure. **d** Postoperative pathological results: GB adenomyomatosis

1.1 Malignant lesions
  Among 34 cases, hyper-, iso-, and hypo-enhancement patterns were observed in 73.5% (25/34), 14.7% (5/34), and 11.8% (4/34) of the cases in the arterial phase, respectively. Among the 25 cases of hyper-enhancement, 20 cases showed heterogeneous enhancement (one case of honeycomb-like enhancement and 19 cases of branch-like enhancement). Among the 34 malignant cases, 31 cases exhibited hypo-enhancement earlier than the adjacent GB wall, whereas the others showed simultaneous wash-out with the GB wall. The mean wash-out time was 30.91 ± 10.40 s (range: 13–60 s). Regarding the pathological results, 15 cases had infiltrated the entire GB wall even the surrounding liver tissue, and 14/15 cases were observed on CEUS. Four cases with liver infiltrated showed the liver tissue a “fast-in and fast-out” enhancement pattern (Fig. 1).
1.2 Cholesterol polyps
  Among 100 cases, five cases (5.0%) showed hyper-enhancement, seven cases (7.0%) showed weak enhancement, and the other 88 cases (88.0%) showed iso-enhancement (81 cases of homogeneous iso-enhancement and seven cases of heterogeneous iso-enhancement) in the arterial phase. In the venous phase, 90 cases (90%) were simultaneously washed out with the GB wall and ten cases showed a shorter wash-out time in comparison to the adjacent GB wall (Fig. 2).
1.3 Adenoma
  In the arterial phase, the findings of 29 cases were similar to those of cholesterol polyps: five cases (17.2%) showed hyper-enhancement, three cases (10.4%) showed weak enhancement, and the other 21 cases (72.4%) showed iso-enhancement (17 cases of homogeneous iso-enhancement and four cases of heterogeneous iso-enhancement). During the venous phase, 22 cases (75.9%) showed a gradual wash-out with the GB wall. However, the other seven cases (24.1%) showed wash-out before the GB wall with the pathological exhibition of active proliferation (Fig. 3).
1.4 Adenomyomatosis
  Among the ten cases, one case (10.0%) showed hyper-enhancement, one case (10.0%) showed weak enhancement, and eight cases (80.0%) showed iso-enhancement (seven cases of honeycomb-like heterogeneous iso-enhancement). In the late phase, synchronous wash-out was observed in eight cases, and the remaining two cases had a shorter wash-out time than the GB wall with pathological confirmation of mild atypical dysplasia (Fig. 4).
1.5 Chronic cholecystitis
  Two cases showed iso-enhancement and one case showed weak enhancement during the arterial phase. In the venous phase, all three cases showed gradual synchronous wash-out with the GB wall.
1.6 Cholecystolithiasis
  Non-enhancement was demonstrated in two cases during the entire CEUS procedure.
1.7 Metaplastic polyp
  This lesion showed synchronous wash-in and wash-out with the GB wall.
1.8 Hyperplastic polyp
  The enhancement of this lesion was slightly slower than that of the GB wall, whereas the enhancement intensity is similar to the GB wall. Then, it showed synchronous wash-out with the GB wall.

**Table 2:** The enhancement extent of ALGs compared to normal GB wall

Final diagnosis	n	Enhancement extent in early phase				Contrast agent wash-out within the lesion		
		Hyper-	Iso-	Hypo-	Non-	Earlier	Same	Later
Malignant lesions	34	25 (73.5%)	5 (14.7%)	4 (11.8%)	0 (0%)	31 (91.2%)	3 (8.8%)	0 (0%)
Benign lesions	146	11 (7.5%)	121(82.9%)	12 (8.2%)	2 (1.4%)	19 (13.2%)	125 (86.8%)	0 (0%)
Cholesterol polyp	100	5 (5.0%)	88 (88.0%)	7 (7.0%)	0 (0%)	10 (10%)	90 (90%)	0 (0%)
Adenoma	29	5 (17.2%)	21 (72.4%)	3 (10.4%)	0 (0%)	7 (24.1%)	22 (75.9%)	0 (0%)
Adenomyomatosis	10	1 (10.0%)	8 (80.0%)	1 (10.0%)	0 (0%)	2 (20.0%)	8 (80.0%)	0 (0%)
Chronic cholecystitis	3	0 (0%)	2 (66.7%)	1 (33.3%)	0 (0%)	0 (0%)	3 (100%)	0 (0%)
Gallstone	2	0 (0%)	0 (0%)	0 (0%)	2 (100%)	–	–	–
Hyperplastic polyp	1	0 (0%)	1 (100%)	0 (0%)	0 (0%)	0 (0%)	1 (100%)	0 (0%)
Metaplastic polyp	1	0 (0%)	1 (100%)	0 (0%)	0 (0%)	0 (0%)	1 (100%)	0 (0%)

^*^There was significant difference between benign and malignant GB diseases in enhancement pattern on CEUS image during both arterial phase and venous phase (arterial phase: χ^2^ = 79.200, *P* = 0.000; venous phase: χ^2^ = 82.808, *P* = 0.000); there was significant difference between cholesterol polyps and adenoma groups in the enhancement pattern of venous phase, χ^2^ = 3.927, *P* = 0.048

2. Statistical analysis of PLGs on CEUS

The mean AT of PLGs of different pathological types was computed, as shown in Table 3. No significant differences were observed in AT between the malignant and benign lesions. Different pathological types of PLGs enhancement patterns are shown in Table 2. The benign lesions (121/146, 82.9%) were mainly iso-enhanced in the arterial phase, whereas the malignant lesions (25/34, 73.5%) were mainly hyper-enhanced. The difference between the two groups was statistically significant (*P* = 0.000). However, no significant differences were observed in enhancement intensity among benign lesions during the arterial phase. In the venous phase, the contrast agent was frequently shown to be synchronously washed out in most benign lesions (125/146, 85.6%) in comparison to the GB wall. The majority of malignant lesions (31/34, 91.2%) typically exhibited wash-out in advance of the GB wall by almost 40 s. The difference in wash-out pattern between malignant and benign lesions indicated statistical significance (*P* = 0.000). Among the benign lesions, the proportion of adenomas showing hypo-enhancement during the venous phase was higher than that of cholesterol polyps, and this difference between the two groups was statistically significant (*P* = 0.048).

**Table 3:** The AT of ALGs of different pathological types

Final diagnosis	n	AT (s)
Malignant lesions	34	16.12 ± 3.61 (10 ~ 26)
Benign lesions	144^a^	15.69 ± 3.04 (9 ~ 24)
Cholesterol polyp	100	15.49 ± 3.00 (9 ~ 20)
Adenoma	29	15.52 ± 2.56 (12 ~ 19)
Adenomyomatosis	10	17.67 ± 4.67 (13 ~ 24)

^a^2 cases of gallstones were excluded. ^*^The measurement data was presented as mean ± SD. The AT between benign groups, as well as the malignant and benign lesions, indicated no statistically significant difference

3. Accuracy of CEUS in the diagnosis of PLGs

The accuracy of CEUS in the diagnosis of PLGs ≥ 1 cm was 92.22% (166/180), and in the diagnosis of benign and malignant PLGs was 92.47% (135/146) and 91.17% (31/34), respectively. The accuracy of different types of PLGs is presented in Table 4. No statistically significant differences were observed between the two groups. When we considered the discontinuity of the GB wall on CEUS images as a standard for malignant lesions that had infiltrated the GB wall or the adjacent hepatic parenchyma, the sensitivity, specificity, and accuracy were 93.33% (14/15), 92.12% (152/165), and 92.22% (166/180), respectively. If we considered the wash-out time ≤ 40 s as the diagnostic standard for malignant lesions, the sensitivity, specificity, and accuracy were 88.24% (30/34), 85.62% (125/146), and 86.11% (155/180), respectively.

**Table 4:** The diagnostic accuracy rate of ALGs

Final diagnosis	n	No. of accuracy diagnosis	Accuracy rate (%)
Malignant lesions^*^	34	31	91.17
Benign lesions	144	135	92.47
Cholesterol polyp^*^	100	93	93.00
Adenoma^*^	29	26	89.66
Adenomyomatosis^*^	10	9	90.00

^*^No statistically significant difference was found between these four types of ALGs in the accuracy rate of CEUS, χ.^2^ = 0.432, *P* = 0.934

## Discussion

**Fig. 5:**
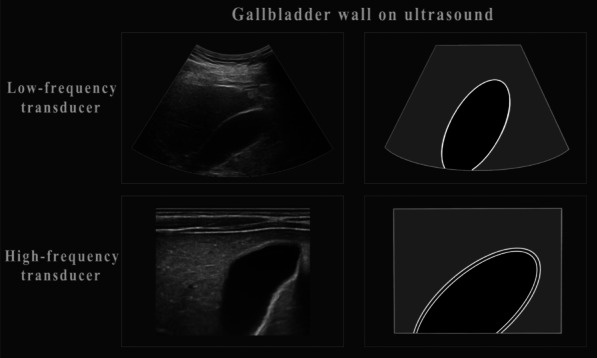
Diagram shows ultrasound (US) appearance of gallbladder (GB) with low-frequency transducer and high-frequency transducer. Low-frequency transducer may depict a single or two layers of gallbladder wall and high-frequency transducer can depict three layers of gallbladder wall: the innermost hyperechoic layer (mucosa), the middle thin hypoechoic layer (muscular) and the outermost hyperechoic layer (serosa)

Conventional US plays a very significant role in the diagnosis of GB diseases [10]. It can clearly appear the normal GB wall. Low-frequency transducers may show a single or two layers of GB wall. High-frequency transducers can show three layers of GB wall: the innermost hyperechoic layer (mucosa), the middle thin hypoechoic layer (muscular) and the outermost hyperechoic layer (serosa) (Fig. 5). In this study, malignant lesions often manifest as multiple, large tumors with a wide base in two-dimensional US images, whereas benign lesions are mostly single, small, and connected by a fine pedicle. Besides, the detection rate of malignant groups by CDFI was much higher.

CEUS can be used to display the whole process of microvascular perfusion in real-time and can improve the differential diagnosis of the disease to the level of microvessels. It has been widely used in the clinical diagnosis of multiple organ diseases, especially in the liver [11]. Zheng’s study [12] showed that CEUS could significantly improve the sensitivity of detection of PLGs (22.2% before CEUS versus 77.8% after CEUS). However, for the PLGs < 1.0 cm, CEUS does not seem to provide any additional benefit. Therefore, CEUS is not recommended for lesions < 1.0 cm.

Because different instruments, imaging software, and contrast agents are used, experts hold various viewpoints on the role of CEUS in the diagnosis of GB diseases. The 2011 EFSUMB non-liver guidelines hold a negative view that there is no diagnostic value in differentiating benign and malignant PLG, because the vascular morphology and enhancement pattern of the lesions are complex in CEUS [13]. Nevertheless, the guidelines suggest CEUS as a valuable tool to determine the presence of hepatic metastasis or destruction of the GB wall. The mainstream view within recent years shows GB lesions on CEUS are different from those of liver lesions, and the enhancement level is of little significance in the differential diagnosis. The CEUS should be more focused on observation of the internal structure of the lesion in the early phase, the disappearance time of the contrast medium, and whether there is infiltration of the GB wall and adjacent liver tissue [6, 7, 10, 13–15].

In our study, except for two cases of gallstones, all the lesions have been enhanced. Benign lesions typically showed rapid and homogeneous enhancement in the arterial phase. No significant differences were noted in the AT between benign and malignant lesions. It could be related to the existence of an abundant arterial blood supply to the GB and is consistent with previous reports [14, 16]. Most of the benign lesions were simultaneously cleared with the GB wall, while the malignant lesions usually showed a “fast-in and fast-out” enhancement pattern. This confirmed the theory that the formation of arteriovenous fistulae and increased blood velocity formed the pathological basis for the rapid clearance of contrast agents, which is consistent with Yuan’s study [17].

In the arterial phase, in comparison to the GB wall enhancement intensity, 82.9% of the benign lesions showed iso-enhancement, whereas 73.5% of malignant lesions were characterized by hyper-enhancement. This difference was statistically significant and showed that the enhancement level was useful to distinguish between benign and malignant GB lesions. However, this result is in contradiction with those of Xie [14] (85% of GB carcinoma and 70% of benign lesions showed hyper-enhancement in the arterial phase), because our study considered the enhancement level of GB wall as a reference, rather than Xie’s adjacent liver tissue. In most cases, the enhancement intensity was compared with that of the liver. The lesion was often found to show synchronous or advanced enhancement against the hepatic parenchyma, presumably because the PLGs were supplied by the biliary artery that arises from the hepatic artery. Theoretically, the enhancement time of PLGs, regardless of whether they are benign or malignant lesions, is either shorter or equal to that of the liver parenchyma [18]. Therefore, hepatic enhancement cannot be considered a reliable standard by which malignant lesions can be identified.

In the venous phase, Xie [14] concluded that 90.9% of GB carcinoma changed from hyper- or iso-enhancement to hypo-enhancement within 35 s, but only 17.0% of benign lesions showed similar characteristics. Liu [15] considered 36.5 s as the diagnostic standard and reported the diagnostic sensitivity and specificity as 74.8% and 49%, respectively. In our study, 67.6% (23/34) of the malignant lesions and 17.8% (26/146) of the benign lesions showed hypo-enhancement within 35 s. The reason why we observed a smaller percentage is that our malignant group included one case of adenomyomatosis canceration and six cases of adenoma canceration which reflected a lower degree of malignancy. Theoretically, the higher the degree of malignancy, the greater the arterial blood supply to the lesion, and the faster the reduction in contrast agent within the lesion. However, If we considered a wash-out time ≤ 40 s as the standard to differentiate malignant and benign lesions, the diagnostic sensitivity, specificity, and accuracy would all be above 85%.

In this study, the proportion of benign adenomas with hypo-enhancement in the venous phase was higher than that of cholesterol polyps. Raica [18] had indicated that the malignancy rate of GB adenoma is about 3.0–58.8%. Kozuka [19] found evidence of pathological change from adenoma to adenoma canceration by discovering the residual adenoma tissue within the pathological section of adenocarcinoma. Therefore, once the GB adenoma is found, it should be actively treated surgically to prevent the occurrence of cancer [20].

In addition, we found that gallbladder adenomyomatosis was characterized by honeycomb-like heterogeneous enhancement in the arterial phase on CEUS. Because there was no enhancement of the Rokitansky–Aschoff sinus in gallbladder adenomyosis during the whole CEUS process.

Our study found that we could use CEUS to observe the continuity of the GB wall and the surrounding liver tissue, with or without infiltration changes, in order to determine the true scope of GB cancer lesions. It can provide a good guide for GB cancer resection range and postoperative evaluation [16, 21, 22].

Against the background of the surgery for PLGs 1 ≥ cm, CEUS is able to get a relatively accurate qualitative diagnosis of most of the lesions before operation. Of the 180 cases in this study, only 63 cases (34 cases of malignant lesions and 29 cases of gallbladder adenoma) really needed surgical treatment. The correct diagnostic rate of CEUS was 90.48% (57/63). The rest 117 could replace surgery by regular re-examination. The correct diagnostic rate of CEUS of these patients was 94.87% (111/117). Therefore, not all PLGs ≥ 1 cm tend to suggest surgical treatment. The surgery should be recommended only if the comprehensive diagnosis of conventional US and CEUS considers a malignant potential lesion. The reduction of cholecystectomies after CEUS evaluation of PLGs ≥ 1 cm can avoid the symptoms of post-cholecystectomy syndrome in these patients, such as right upper quadrant abdominal pain, dyspepsia, and/or jaundice, and reduce the psychological and economic burden of patients and their families to a certain extent.

In conclusion, this study confirmed that CEUS is valuable in the differential diagnosis of malignant and benign PLGs. The “fast-in and fast-out” enhancement pattern, higher enhancement level in comparison to the GB wall in the arterial phase, wash-out time ≤ 40 s, wall destruction, and hepatic parenchyma infiltration are frequently encountered in malignant PLGs. However, accurate identification of benign PLGs of different pathological types on CEUS seems challenging. In addition, conventional US is also important in reading CEUS images. Malignancies were more likely to be diagnosed if the lesion was found to be larger, single, sessile, and with a detectable blood flow signal on the conventional US. The major limitation of this study is that the accuracy of conventional ultrasound is not compared with that of CEUS. The second limitation was the relatively small number of GB carcinoma detected, but which could be mostly attributed to its low incidence. Thus, further study of GB carcinoma with a larger number of cases is required.

## Data Availability

The datasets used and/or analysed during the current study available from the corresponding author on reasonable.

## Abbreviations

AT: Arrival time
CDFI: Color Doppler flow imaging
CECT: Contrast-enhanced computer tomography
CEUS: Contrast-enhanced ultrasound
GB: Gallbladder
MI: Mechanical index
MRCP: Magnetic resonance cholangiopancreatography
PLGs: Polypoid lesions of gallbladder
US: Ultrasonography
